# Practical Ontologies for Information Professionals

**DOI:** 10.5195/jmla.2017.338

**Published:** 2017-10-01

**Authors:** Suzanne Duncan

This book, while not lengthy, is packed with a wealth of information about ontologies. It is broken into seven chapters that progress in a logical manner, starting with what an ontology is and covering how ontologies have developed with the need to handle data and information overload. The parts, types, and language of ontologies are discussed. The first chapter is a practical introduction to ontologies. The reader is then introduced to ontologies and the semantic web. Resources—such as web ontology language (OWL), resource description framework schema (RDFS), and SPARQL, a resource description framework (RDF) query language—that are needed to structure and search information are presented.

Next in the flow are existing ontologies and adopting ontologies. Each of these chapters covers many of the more popular ontologies, for example, Dublin Core, simple knowledge organization systems (SKOS), OWL2, and functional requirements for bibliographic records (FRBR). Strengths and weakness are then reviewed. Examples of possible uses and values for each are also covered. The levels of ontologies (upper level, non-domain-specific, and frameworks) add to the image of the complexity but would be familiar to an information professional who works with structure and retrieval of information. Ontology libraries, semantic search engines, and web search engines are included in the conversation of how to find the right ontology or determine if you need to create your own.

The longest chapter in the book is all about building ontologies. I would consider this the meat of the book. David Stuart presents twelve steps in the process of creating an ontology, each of which is discussed in depth. I have worked with information technology staff on creating ontologies and using existing ontologies.

Stuart, in my opinion, really got the steps right. The most important is step 1: what the scope is, clearly defining the audience and bounds of the ontology that you are about to create, making it easier to stay on point. Additionally, steps eleven (documentation) and twelve (maintenance and sustainability) are as important as the previous steps but can be lost in the process once an ontology’s initial creation is “completed,” based on my personal experience and discussing with other information professionals.

The chapter concludes with a walk-through using the twelve steps in creating an ontology. The questions and decisions needed in each step are presented during the development of this example ontology. There is a uniform resource locator (URL) that goes to the created ontology for the reader to see the completed project.

Interrogating—which is exploring or interrogating ontologies for reuse, understanding how they are used or how to pull information from the data in the ontology, and the tools needed for that process—is in the next chapter. This is important to help determine if the ontology is working the way that you want and what “new” or different information an ontology allows you to find.

The book finishes with a look to the future for information professionals and ontologies. This chapter provides a look at future ontologies and ways to determine when some other tool might be better for your project’s discovery needs. Ontologies are another way to look at and retrieve information to gain new insights, especially with vast quantities of data. The role of the information professional in developing ontologies in libraries and other settings is presented by Stuart.

My first project creating an ontology was not for the library but rather for the intranet in my organization. I found the examples, websites, and practical information presented in this book to be interesting and valuable. I am acquiring a copy of this book to give to the information technology team that I work with on ontologies. I found myself many times, during the current project that we are working on, mentioning items and resources in the book. While focusing on information professionals, this book will be an excellent resource for any individual working on an ontology or information discovery project.

